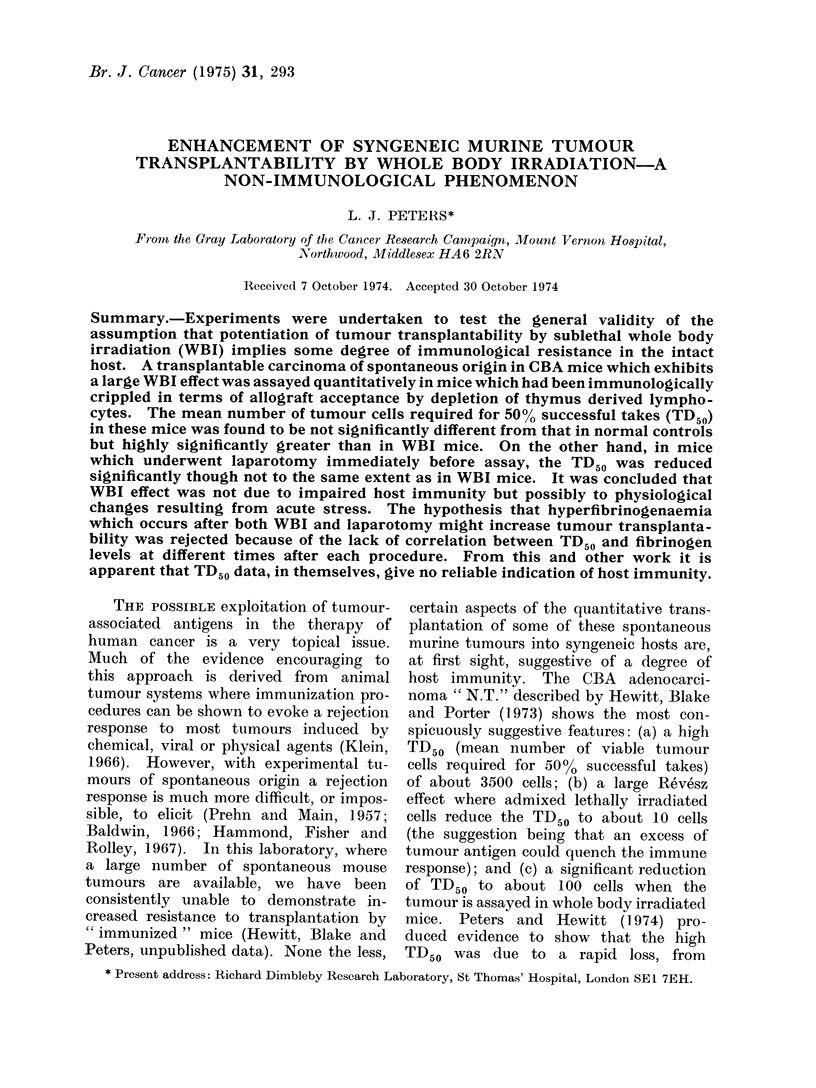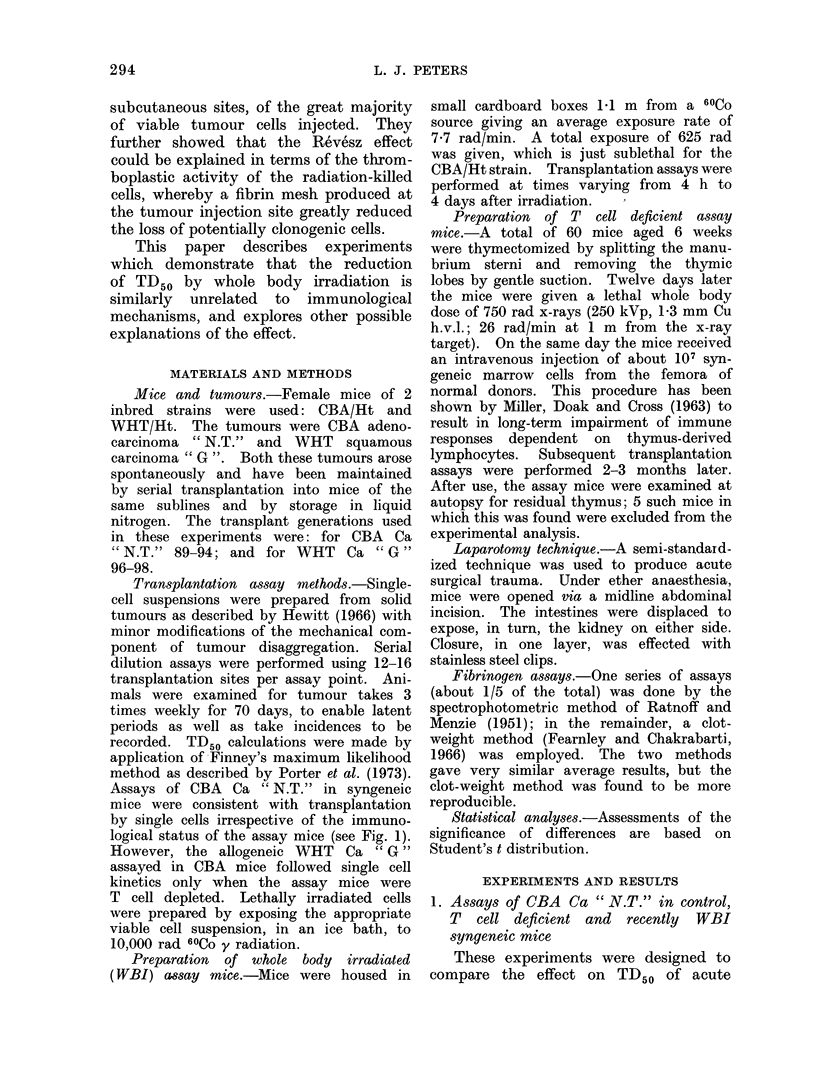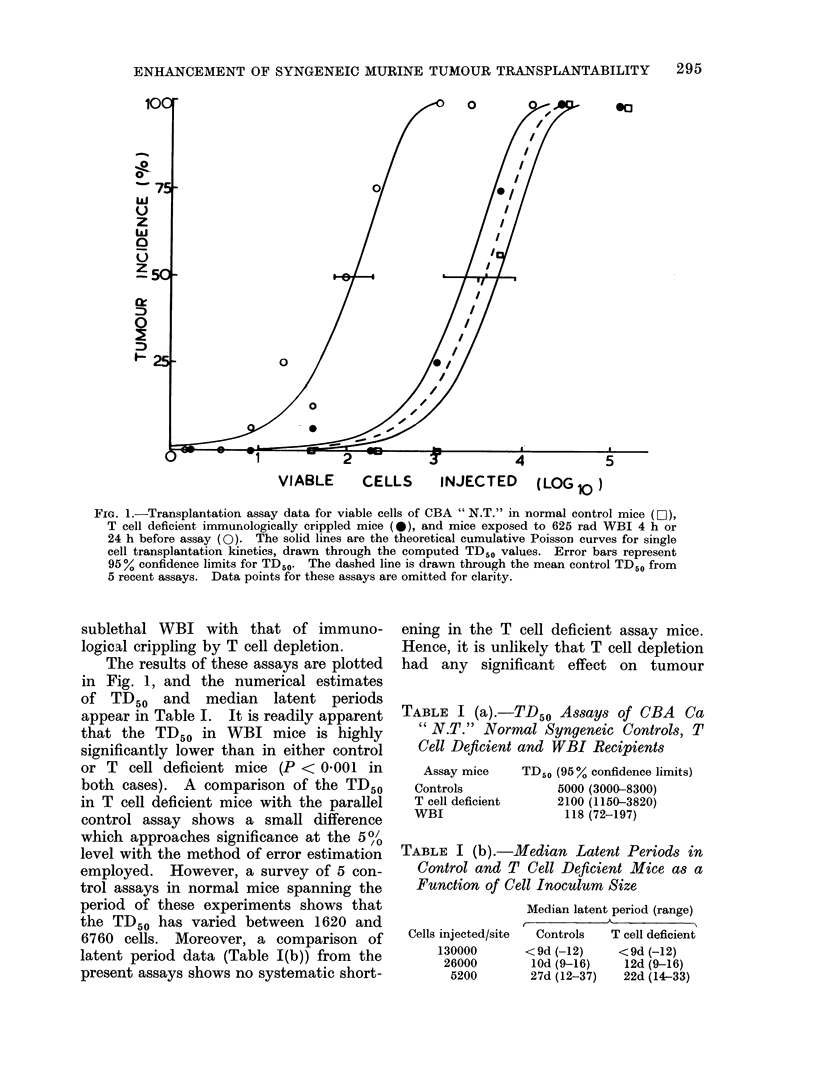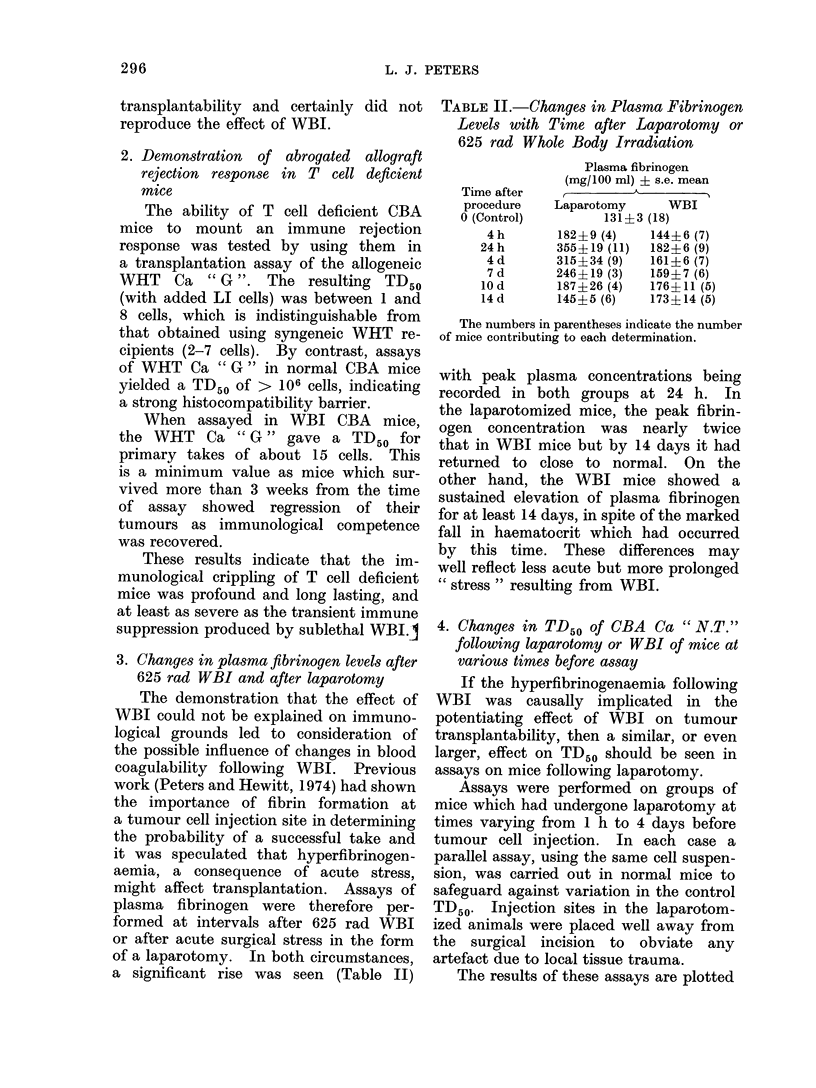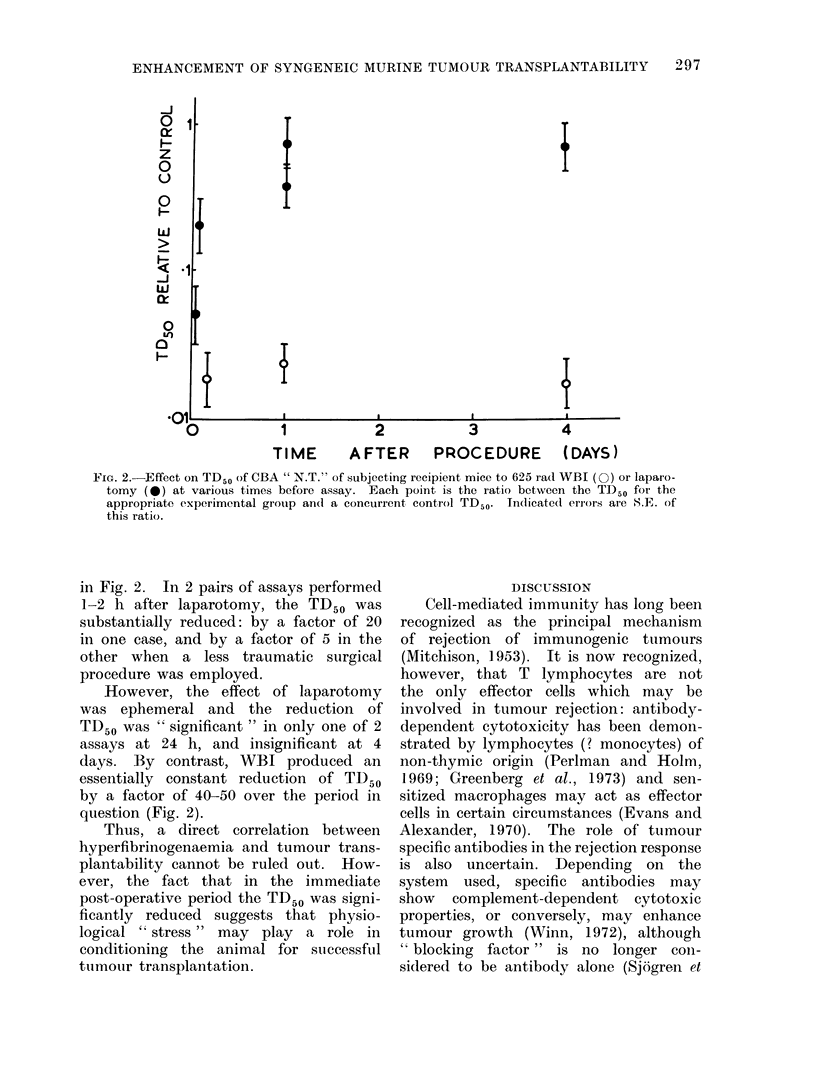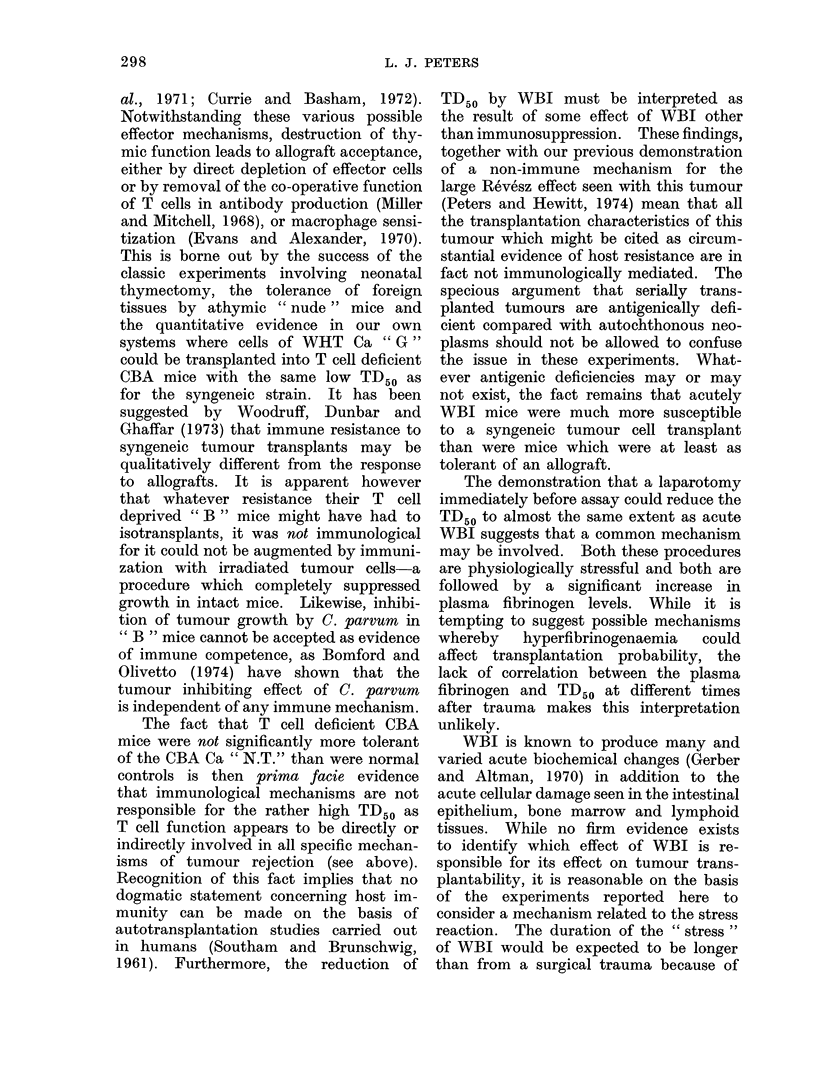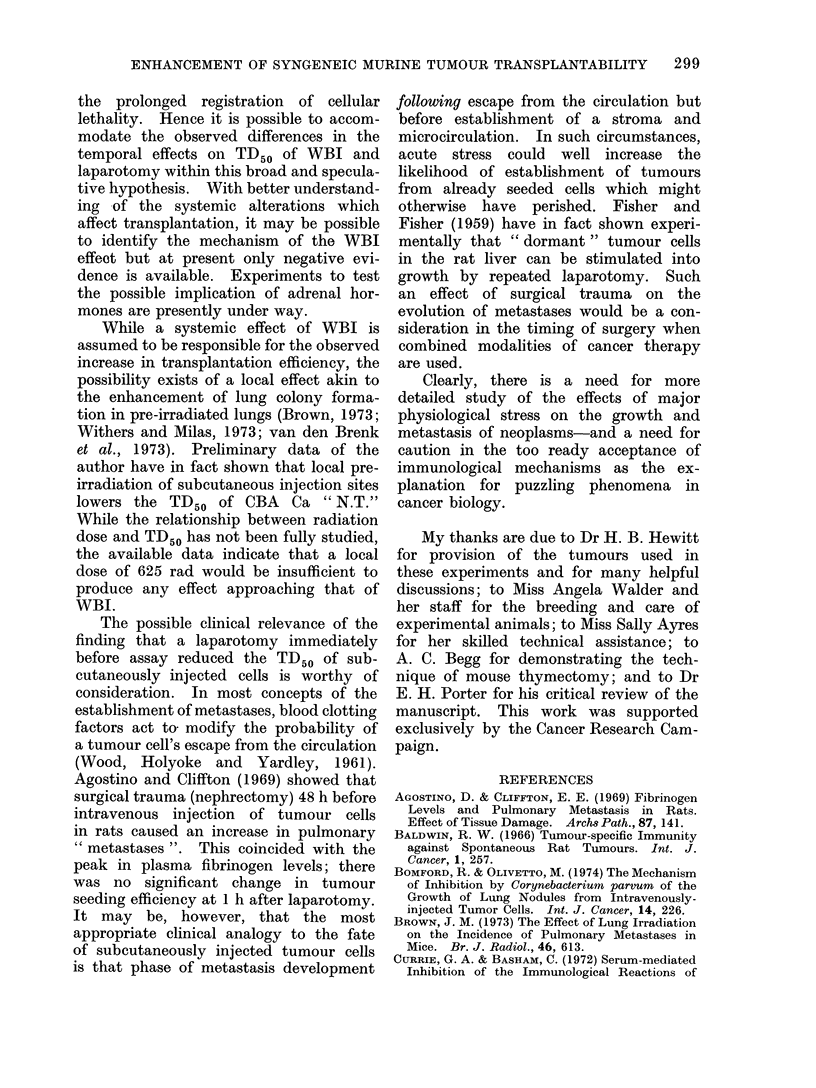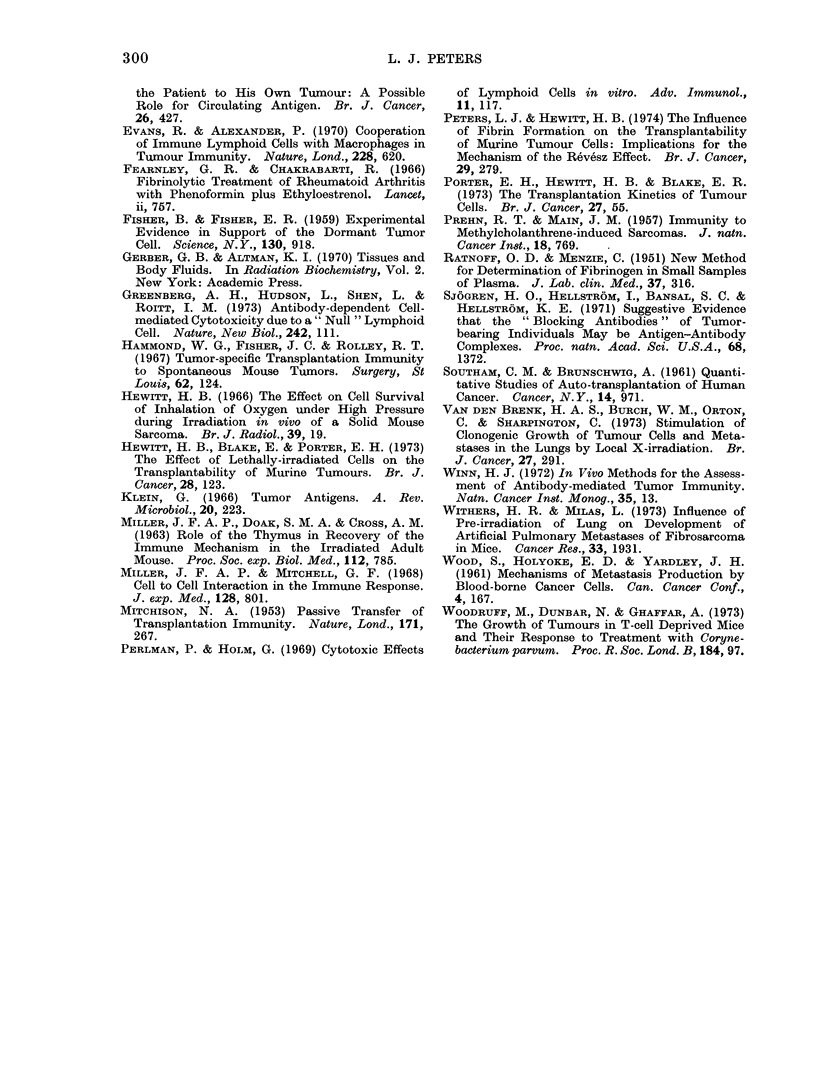# Enhancement of syngeneic murine tumour transplantability by whole body irradiation--a non-immunological phenomenon.

**DOI:** 10.1038/bjc.1975.64

**Published:** 1975-03

**Authors:** L. J. Peters

## Abstract

Experiments were undertaken to test the general validity of the assumption that potentiation of tumour transplantability by sublethal whole body irradiation (WBI) implies some degree of immunological resistance in the intact host. A transplantable carcinoma of spontaneous origin in CBA mice which exhibits a large WBI effect was assayed quantitatively in mice which had been immunologically crippled in terms of allograft acceptance by depletion of thymus derived lymphocytes. The mean number of tumour cells required for 50% successful takes (TD50) in these mice was found to be not significantly different from that in normal controls but highly significantly greater than in WBI mice. On the other hand, in mice which underwent laparotomy immediately before assay, the TD50 was reduced significantly though not to the same extent as in WBI mice. It was concluded that WBI effect was not due to impaired host immunity but possibly to physiological changes resulting from acute stress. The hypothesis that hyperfibrinogenaemia which occurs after both WBI and laparotomy might increase tumour transplantability was rejected because of the lack of correlation between TD50 and fibrinogen levels at different times after each procedure. From this and other work it is apparent that TD50 data, in themselves, give no reliable indication of host immunity.


*Br. J. Cancer* (1975) **31**, 293

### ENHANCEMENT OF SYNGENEIC MURINE TUMOUR TRANSPLANTABILITY BY WHOLE BODY IRRADIATION—A NON-IMMUNOLOGICAL PHENOMENON

L. J. PETERS*

*From the Gray Laboratory of the Cancer Research Campaign, Mount Vernon Hospital, Northwood, Middlesex HA6 2RN*

Received 7 October 1974. Accepted 30 October 1974

**Summary.—Experiments were undertaken to test the general validity of the assumption that potentiation of tumour transplantability by sublethal whole body irradiation (WBI) implies some degree of immunological resistance in the intact host. A transplantable carcinoma of spontaneous origin in CBA mice which exhibits a large WBI effect was assayed quantitatively in mice which had been immunologically crippled in terms of allograft acceptance by depletion of thymus derived lymphocytes. The mean number of tumour cells required for 50% successful takes ($TD_{50}$) in these mice was found to be not significantly different from that in normal controls but highly significantly greater than in WBI mice. On the other hand, in mice which underwent laparotomy immediately before assay, the $TD_{50}$ was reduced significantly though not to the same extent as in WBI mice. It was concluded that WBI effect was not due to impaired host immunity but possibly to physiological changes resulting from acute stress. The hypothesis that hyperfibrinogenaemia which occurs after both WBI and laparotomy might increase tumour transplantability was rejected because of the lack of correlation between $TD_{50}$ and fibrinogen levels at different times after each procedure. From this and other work it is apparent that $TD_{50}$ data, in themselves, give no reliable indication of host immunity.**

THE POSSIBLE exploitation of tumour-associated antigens in the therapy of human cancer is a very topical issue. Much of the evidence encouraging to this approach is derived from animal tumour systems where immunization procedures can be shown to evoke a rejection response to most tumours induced by chemical, viral or physical agents (Klein, 1966). However, with experimental tumours of spontaneous origin a rejection response is much more difficult, or impossible, to elicit (Prehn and Main, 1957; Baldwin, 1966; Hammond, Fisher and Rolley, 1967). In this laboratory, where a large number of spontaneous mouse tumours are available, we have been consistently unable to demonstrate increased resistance to transplantation by "immunized" mice (Hewitt, Blake and Peters, unpublished data). None the less, certain aspects of the quantitative transplantation of some of these spontaneous murine tumours into syngeneic hosts are, at first sight, suggestive of a degree of host immunity. The CBA adenocarcinoma "N.T." described by Hewitt, Blake and Porter (1973) shows the most conspicuously suggestive features: (a) a high $TD_{50}$ (mean number of viable tumour cells required for 50% successful takes) of about 3500 cells; (b) a large Révész effect where admixed lethally irradiated cells reduce the $TD_{50}$ to about 10 cells (the suggestion being that an excess of tumour antigen could quench the immune response); and (c) a significant reduction of $TD_{50}$ to about 100 cells when the tumour is assayed in whole body irradiated mice. Peters and Hewitt (1974) produced evidence to show that the high $TD_{50}$ was due to a rapid loss, from

* Present address: Richard Dimbleby Research Laboratory, St Thomas' Hospital, London SE1 7EH.

subcutaneous sites, of the great majority of viable tumour cells injected. They further showed that the Révész effect could be explained in terms of the thromboplastic activity of the radiation-killed cells, whereby a fibrin mesh produced at the tumour injection site greatly reduced the loss of potentially clonogenic cells.

This paper describes experiments which demonstrate that the reduction of $TD_{50}$ by whole body irradiation is similarly unrelated to immunological mechanisms, and explores other possible explanations of the effect.

### MATERIALS AND METHODS

*Mice and tumours.*—Female mice of 2 inbred strains were used: CBA/Ht and WHT/Ht. The tumours were CBA adenocarcinoma " N.T." and WHT squamous carcinoma " G ". Both these tumours arose spontaneously and have been maintained by serial transplantation into mice of the same sublines and by storage in liquid nitrogen. The transplant generations used in these experiments were: for CBA Ca " N.T." 89–94; and for WHT Ca " G " 96–98.

*Transplantation assay methods.*—Single-cell suspensions were prepared from solid tumours as described by Hewitt (1966) with minor modifications of the mechanical component of tumour disaggregation. Serial dilution assays were performed using 12–16 transplantation sites per assay point. Animals were examined for tumour takes 3 times weekly for 70 days, to enable latent periods as well as take incidences to be recorded. $TD_{50}$ calculations were made by application of Finney's maximum likelihood method as described by Porter *et al.* (1973). Assays of CBA Ca " N.T." in syngeneic mice were consistent with transplantation by single cells irrespective of the immunological status of the assay mice (see Fig. 1). However, the allogeneic WHT Ca " G " assayed in CBA mice followed single cell kinetics only when the assay mice were T cell depleted. Lethally irradiated cells were prepared by exposing the appropriate viable cell suspension, in an ice bath, to 10,000 rad $^{60}Co$ $\gamma$ radiation.

*Preparation of whole body irradiated (WBI) assay mice.*—Mice were housed in small cardboard boxes 1·1 m from a $^{60}Co$ source giving an average exposure rate of 7·7 rad/min. A total exposure of 625 rad was given, which is just sublethal for the CBA/Ht strain. Transplantation assays were performed at times varying from 4 h to 4 days after irradiation.

*Preparation of T cell deficient assay mice.*—A total of 60 mice aged 6 weeks were thymectomized by splitting the manubrium sterni and removing the thymic lobes by gentle suction. Twelve days later the mice were given a lethal whole body dose of 750 rad x-rays (250 kVp, 1·3 mm Cu h.v.l.; 26 rad/min at 1 m from the x-ray target). On the same day the mice received an intravenous injection of about $10^7$ syngeneic marrow cells from the femora of normal donors. This procedure has been shown by Miller, Doak and Cross (1963) to result in long-term impairment of immune responses dependent on thymus-derived lymphocytes. Subsequent transplantation assays were performed 2–3 months later. After use, the assay mice were examined at autopsy for residual thymus; 5 such mice in which this was found were excluded from the experimental analysis.

*Laparotomy technique.*—A semi-standardized technique was used to produce acute surgical trauma. Under ether anaesthesia, mice were opened *via* a midline abdominal incision. The intestines were displaced to expose, in turn, the kidney on either side. Closure, in one layer, was effected with stainless steel clips.

*Fibrinogen assays.*—One series of assays (about 1/5 of the total) was done by the spectrophotometric method of Ratnoff and Menzie (1951); in the remainder, a clot-weight method (Fearnley and Chakrabarti, 1966) was employed. The two methods gave very similar average results, but the clot-weight method was found to be more reproducible.

*Statistical analyses.*—Assessments of the significance of differences are based on Student's $t$ distribution.

### EXPERIMENTS AND RESULTS

### 1. *Assays of CBA Ca " N.T." in control, T cell deficient and recently WBI syngeneic mice*

These experiments were designed to compare the effect on $TD_{50}$ of acute

FIG. 1.—Transplantation assay data for viable cells of CBA " N.T." in normal control mice (□), T cell deficient immunologically crippled mice (●), and mice exposed to 625 rad WBI 4 h or 24 h before assay (○). The solid lines are the theoretical cumulative Poisson curves for single cell transplantation kinetics, drawn through the computed $TD_{50}$ values. Error bars represent 95% confidence limits for $TD_{50}$. The dashed line is drawn through the mean control $TD_{50}$ from 5 recent assays. Data points for these assays are omitted for clarity.

sublethal WBI with that of immunological crippling by T cell depletion.

The results of these assays are plotted in Fig. 1, and the numerical estimates of $TD_{50}$ and median latent periods appear in Table I. It is readily apparent that the $TD_{50}$ in WBI mice is highly significantly lower than in either control or T cell deficient mice ($P < 0{\cdot}001$ in both cases). A comparison of the $TD_{50}$ in T cell deficient mice with the parallel control assay shows a small difference which approaches significance at the 5% level with the method of error estimation employed. However, a survey of 5 control assays in normal mice spanning the period of these experiments shows that the $TD_{50}$ has varied between 1620 and 6760 cells. Moreover, a comparison of latent period data (Table I(b)) from the present assays shows no systematic shortening in the T cell deficient assay mice. Hence, it is unlikely that T cell depletion had any significant effect on tumour

TABLE I (a).—*$TD_{50}$ Assays of CBA Ca " N.T." Normal Syngeneic Controls, T Cell Deficient and WBI Recipients*

| Assay mice | $TD_{50}$ (95% confidence limits) |
|---|---|
| Controls | 5000 (3000–8300) |
| T cell deficient | 2100 (1150–3820) |
| WBI | 118 (72–197) |

TABLE I (b).—*Median Latent Periods in Control and T Cell Deficient Mice as a Function of Cell Inoculum Size*

| | Median latent period (range) | |
|---|---|---|
| Cells injected/site | Controls | T cell deficient |
| 130000 | <9d (–12) | <9d (–12) |
| 26000 | 10d (9–16) | 12d (9–16) |
| 5200 | 27d (12–37) | 22d (14–33) |

transplantability and certainly did not reproduce the effect of WBI.

2. *Demonstration of abrogated allograft rejection response in T cell deficient mice*

The ability of T cell deficient CBA mice to mount an immune rejection response was tested by using them in a transplantation assay of the allogeneic WHT Ca "G". The resulting $TD_{50}$ (with added LI cells) was between 1 and 8 cells, which is indistinguishable from that obtained using syngeneic WHT recipients (2–7 cells). By contrast, assays of WHT Ca "G" in normal CBA mice yielded a $TD_{50}$ of $> 10^6$ cells, indicating a strong histocompatibility barrier.

When assayed in WBI CBA mice, the WHT Ca "G" gave a $TD_{50}$ for primary takes of about 15 cells. This is a minimum value as mice which survived more than 3 weeks from the time of assay showed regression of their tumours as immunological competence was recovered.

These results indicate that the immunological crippling of T cell deficient mice was profound and long lasting, and at least as severe as the transient immune suppression produced by sublethal WBI.

3. *Changes in plasma fibrinogen levels after 625 rad WBI and after laparotomy*

The demonstration that the effect of WBI could not be explained on immunological grounds led to consideration of the possible influence of changes in blood coagulability following WBI. Previous work (Peters and Hewitt, 1974) had shown the importance of fibrin formation at a tumour cell injection site in determining the probability of a successful take and it was speculated that hyperfibrinogenaemia, a consequence of acute stress, might affect transplantation. Assays of plasma fibrinogen were therefore performed at intervals after 625 rad WBI or after acute surgical stress in the form of a laparotomy. In both circumstances, a significant rise was seen (Table II)

TABLE II.—*Changes in Plasma Fibrinogen Levels with Time after Laparotomy or 625 rad Whole Body Irradiation*

| Time after procedure | Plasma fibrinogen (mg/100 ml) ± s.e. mean: Laparotomy | WBI |
|---|---|---|
| 0 (Control) | 131±3 (18) | |
| 4 h | 182±9 (4) | 144±6 (7) |
| 24 h | 355±19 (11) | 182±6 (9) |
| 4 d | 315±34 (9) | 161±6 (7) |
| 7 d | 246±19 (3) | 159±7 (6) |
| 10 d | 187±26 (4) | 176±11 (5) |
| 14 d | 145±5 (6) | 173±14 (5) |

The numbers in parentheses indicate the number of mice contributing to each determination.

with peak plasma concentrations being recorded in both groups at 24 h. In the laparotomized mice, the peak fibrinogen concentration was nearly twice that in WBI mice but by 14 days it had returned to close to normal. On the other hand, the WBI mice showed a sustained elevation of plasma fibrinogen for at least 14 days, in spite of the marked fall in haematocrit which had occurred by this time. These differences may well reflect less acute but more prolonged "stress" resulting from WBI.

4. *Changes in $TD_{50}$ of CBA Ca "N.T." following laparotomy or WBI of mice at various times before assay*

If the hyperfibrinogenaemia following WBI was causally implicated in the potentiating effect of WBI on tumour transplantability, then a similar, or even larger, effect on $TD_{50}$ should be seen in assays on mice following laparotomy.

Assays were performed on groups of mice which had undergone laparotomy at times varying from 1 h to 4 days before tumour cell injection. In each case a parallel assay, using the same cell suspension, was carried out in normal mice to safeguard against variation in the control $TD_{50}$. Injection sites in the laparotomized animals were placed well away from the surgical incision to obviate any artefact due to local tissue trauma.

The results of these assays are plotted

FIG. 2.—Effect on $TD_{50}$ of CBA " N.T." of subjecting recipient mice to 625 rad WBI (○) or laparotomy (●) at various times before assay. Each point is the ratio between the $TD_{50}$ for the appropriate experimental group and a concurrent control $TD_{50}$. Indicated errors are S.E. of this ratio.

in Fig. 2. In 2 pairs of assays performed 1–2 h after laparotomy, the $TD_{50}$ was substantially reduced: by a factor of 20 in one case, and by a factor of 5 in the other when a less traumatic surgical procedure was employed.

However, the effect of laparotomy was ephemeral and the reduction of $TD_{50}$ was " significant " in only one of 2 assays at 24 h, and insignificant at 4 days. By contrast, WBI produced an essentially constant reduction of $TD_{50}$ by a factor of 40–50 over the period in question (Fig. 2).

Thus, a direct correlation between hyperfibrinogenaemia and tumour transplantability cannot be ruled out. However, the fact that in the immediate post-operative period the $TD_{50}$ was significantly reduced suggests that physiological " stress " may play a role in conditioning the animal for successful tumour transplantation.

DISCUSSION

Cell-mediated immunity has long been recognized as the principal mechanism of rejection of immunogenic tumours (Mitchison, 1953). It is now recognized, however, that T lymphocytes are not the only effector cells which may be involved in tumour rejection: antibody-dependent cytotoxicity has been demonstrated by lymphocytes (? monocytes) of non-thymic origin (Perlman and Holm, 1969; Greenberg *et al.*, 1973) and sensitized macrophages may act as effector cells in certain circumstances (Evans and Alexander, 1970). The role of tumour specific antibodies in the rejection response is also uncertain. Depending on the system used, specific antibodies may show complement-dependent cytotoxic properties, or conversely, may enhance tumour growth (Winn, 1972), although " blocking factor " is no longer considered to be antibody alone (Sjögren *et*

*al.*, 1971; Currie and Basham, 1972). Notwithstanding these various possible effector mechanisms, destruction of thymic function leads to allograft acceptance, either by direct depletion of effector cells or by removal of the co-operative function of T cells in antibody production (Miller and Mitchell, 1968), or macrophage sensitization (Evans and Alexander, 1970). This is borne out by the success of the classic experiments involving neonatal thymectomy, the tolerance of foreign tissues by athymic " nude " mice and the quantitative evidence in our own systems where cells of WHT Ca " G " could be transplanted into T cell deficient CBA mice with the same low $TD_{50}$ as for the syngeneic strain. It has been suggested by Woodruff, Dunbar and Ghaffar (1973) that immune resistance to syngeneic tumour transplants may be qualitatively different from the response to allografts. It is apparent however that whatever resistance their T cell deprived " B " mice might have had to isotransplants, it was *not* immunological for it could not be augmented by immunization with irradiated tumour cells—a procedure which completely suppressed growth in intact mice. Likewise, inhibition of tumour growth by *C. parvum* in " B " mice cannot be accepted as evidence of immune competence, as Bomford and Olivetto (1974) have shown that the tumour inhibiting effect of *C. parvum* is independent of any immune mechanism.

The fact that T cell deficient CBA mice were *not* significantly more tolerant of the CBA Ca " N.T." than were normal controls is then *prima facie* evidence that immunological mechanisms are not responsible for the rather high $TD_{50}$ as T cell function appears to be directly or indirectly involved in all specific mechanisms of tumour rejection (see above). Recognition of this fact implies that no dogmatic statement concerning host immunity can be made on the basis of autotransplantation studies carried out in humans (Southam and Brunschwig, 1961). Furthermore, the reduction of $TD_{50}$ by WBI must be interpreted as the result of some effect of WBI other than immunosuppression. These findings, together with our previous demonstration of a non-immune mechanism for the large Révész effect seen with this tumour (Peters and Hewitt, 1974) mean that all the transplantation characteristics of this tumour which might be cited as circumstantial evidence of host resistance are in fact not immunologically mediated. The specious argument that serially transplanted tumours are antigenically deficient compared with autochthonous neoplasms should not be allowed to confuse the issue in these experiments. Whatever antigenic deficiencies may or may not exist, the fact remains that acutely WBI mice were much more susceptible to a syngeneic tumour cell transplant than were mice which were at least as tolerant of an allograft.

The demonstration that a laparotomy immediately before assay could reduce the $TD_{50}$ to almost the same extent as acute WBI suggests that a common mechanism may be involved. Both these procedures are physiologically stressful and both are followed by a significant increase in plasma fibrinogen levels. While it is tempting to suggest possible mechanisms whereby hyperfibrinogenaemia could affect transplantation probability, the lack of correlation between the plasma fibrinogen and $TD_{50}$ at different times after trauma makes this interpretation unlikely.

WBI is known to produce many and varied acute biochemical changes (Gerber and Altman, 1970) in addition to the acute cellular damage seen in the intestinal epithelium, bone marrow and lymphoid tissues. While no firm evidence exists to identify which effect of WBI is responsible for its effect on tumour transplantability, it is reasonable on the basis of the experiments reported here to consider a mechanism related to the stress reaction. The duration of the " stress " of WBI would be expected to be longer than from a surgical trauma because of the prolonged registration of cellular lethality. Hence it is possible to accommodate the observed differences in the temporal effects on $TD_{50}$ of WBI and laparotomy within this broad and speculative hypothesis. With better understanding of the systemic alterations which affect transplantation, it may be possible to identify the mechanism of the WBI effect but at present only negative evidence is available. Experiments to test the possible implication of adrenal hormones are presently under way.

While a systemic effect of WBI is assumed to be responsible for the observed increase in transplantation efficiency, the possibility exists of a local effect akin to the enhancement of lung colony formation in pre-irradiated lungs (Brown, 1973; Withers and Milas, 1973; van den Brenk *et al.*, 1973). Preliminary data of the author have in fact shown that local pre-irradiation of subcutaneous injection sites lowers the $TD_{50}$ of CBA Ca "N.T." While the relationship between radiation dose and $TD_{50}$ has not been fully studied, the available data indicate that a local dose of 625 rad would be insufficient to produce any effect approaching that of WBI.

The possible clinical relevance of the finding that a laparotomy immediately before assay reduced the $TD_{50}$ of subcutaneously injected cells is worthy of consideration. In most concepts of the establishment of metastases, blood clotting factors act to modify the probability of a tumour cell's escape from the circulation (Wood, Holyoke and Yardley, 1961). Agostino and Cliffton (1969) showed that surgical trauma (nephrectomy) 48 h before intravenous injection of tumour cells in rats caused an increase in pulmonary "metastases". This coincided with the peak in plasma fibrinogen levels; there was no significant change in tumour seeding efficiency at 1 h after laparotomy. It may be, however, that the most appropriate clinical analogy to the fate of subcutaneously injected tumour cells is that phase of metastasis development *following* escape from the circulation but before establishment of a stroma and microcirculation. In such circumstances, acute stress could well increase the likelihood of establishment of tumours from already seeded cells which might otherwise have perished. Fisher and Fisher (1959) have in fact shown experimentally that "dormant" tumour cells in the rat liver can be stimulated into growth by repeated laparotomy. Such an effect of surgical trauma on the evolution of metastases would be a consideration in the timing of surgery when combined modalities of cancer therapy are used.

Clearly, there is a need for more detailed study of the effects of major physiological stress on the growth and metastasis of neoplasms—and a need for caution in the too ready acceptance of immunological mechanisms as the explanation for puzzling phenomena in cancer biology.

My thanks are due to Dr H. B. Hewitt for provision of the tumours used in these experiments and for many helpful discussions; to Miss Angela Walder and her staff for the breeding and care of experimental animals; to Miss Sally Ayres for her skilled technical assistance; to A. C. Begg for demonstrating the technique of mouse thymectomy; and to Dr E. H. Porter for his critical review of the manuscript. This work was supported exclusively by the Cancer Research Campaign.

### REFERENCES

AGOSTINO, D. & CLIFFTON, E. E. (1969) Fibrinogen Levels and Pulmonary Metastasis in Rats. Effect of Tissue Damage. *Archs Path.*, **87**, 141.

BALDWIN, R. W. (1966) Tumour-specific Immunity against Spontaneous Rat Tumours. *Int. J. Cancer*, **1**, 257.

BOMFORD, R. & OLIVETTO, M. (1974) The Mechanism of Inhibition by *Corynebacterium parvum* of the Growth of Lung Nodules from Intravenously-injected Tumor Cells. *Int. J. Cancer*, **14**, 226.

BROWN, J. M. (1973) The Effect of Lung Irradiation on the Incidence of Pulmonary Metastases in Mice. *Br. J. Radiol.*, **46**, 613.

CURRIE, G. A. & BASHAM, C. (1972) Serum-mediated Inhibition of the Immunological Reactions of the Patient to His Own Tumour: A Possible Role for Circulating Antigen. *Br. J. Cancer*, **26**, 427.

EVANS, R. & ALEXANDER, P. (1970) Cooperation of Immune Lymphoid Cells with Macrophages in Tumour Immunity. *Nature, Lond.*, **228**, 620.

FEARNLEY, G. R. & CHAKRABARTI, R. (1966) Fibrinolytic Treatment of Rheumatoid Arthritis with Phenoformin plus Ethyloestrenol. *Lancet*, ii, 757.

FISHER, B. & FISHER, E. R. (1959) Experimental Evidence in Support of the Dormant Tumor Cell. *Science, N.Y.*, **130**, 918.

GERBER, G. B. & ALTMAN, K. I. (1970) Tissues and Body Fluids. In *Radiation Biochemistry*, Vol. 2. New York: Academic Press.

GREENBERG, A. H., HUDSON, L., SHEN, L. & ROITT, I. M. (1973) Antibody-dependent Cell-mediated Cytotoxicity due to a "Null" Lymphoid Cell. *Nature, New Biol.*, **242**, 111.

HAMMOND, W. G., FISHER, J. C. & ROLLEY, R. T. (1967) Tumor-specific Transplantation Immunity to Spontaneous Mouse Tumors. *Surgery, St Louis*, **62**, 124.

HEWITT, H. B. (1966) The Effect on Cell Survival of Inhalation of Oxygen under High Pressure during Irradiation *in vivo* of a Solid Mouse Sarcoma. *Br. J. Radiol.*, **39**, 19.

HEWITT, H. B., BLAKE, E. & PORTER, E. H. (1973) The Effect of Lethally-irradiated Cells on the Transplantability of Murine Tumours. *Br. J. Cancer*, **28**, 123.

KLEIN, G. (1966) Tumor Antigens. *A. Rev. Microbiol.*, **20**, 223.

MILLER, J. F. A. P., DOAK, S. M. A. & CROSS, A. M. (1963) Role of the Thymus in Recovery of the Immune Mechanism in the Irradiated Adult Mouse. *Proc. Soc. exp. Biol. Med.*, **112**, 785.

MILLER, J. F. A. P. & MITCHELL, G. F. (1968) Cell to Cell Interaction in the Immune Response. *J. exp. Med.*, **128**, 801.

MITCHISON, N. A. (1953) Passive Transfer of Transplantation Immunity. *Nature, Lond.*, **171**, 267.

PERLMAN, P. & HOLM, G. (1969) Cytotoxic Effects of Lymphoid Cells *in vitro*. *Adv. Immunol.*, **11**, 117.

PETERS, L. J. & HEWITT, H. B. (1974) The Influence of Fibrin Formation on the Transplantability of Murine Tumour Cells: Implications for the Mechanism of the Révész Effect. *Br. J. Cancer*, **29**, 279.

PORTER, E. H., HEWITT, H. B. & BLAKE, E. R. (1973) The Transplantation Kinetics of Tumour Cells. *Br. J. Cancer*, **27**, 55.

PREHN, R. T. & MAIN, J. M. (1957) Immunity to Methylcholanthrene-induced Sarcomas. *J. natn. Cancer Inst.*, **18**, 769.

RATNOFF, O. D. & MENZIE, C. (1951) New Method for Determination of Fibrinogen in Small Samples of Plasma. *J. Lab. clin. Med.*, **37**, 316.

SJÖGREN, H. O., HELLSTRÖM, I., BANSAL, S. C. & HELLSTRÖM, K. E. (1971) Suggestive Evidence that the "Blocking Antibodies" of Tumor-bearing Individuals May be Antigen–Antibody Complexes. *Proc. natn. Acad. Sci. U.S.A.*, **68**, 1372.

SOUTHAM, C. M. & BRUNSCHWIG, A. (1961) Quantitative Studies of Auto-transplantation of Human Cancer. *Cancer, N.Y.*, **14**, 971.

VAN DEN BRENK, H. A. S., BURCH, W. M., ORTON, C. & SHARPINGTON, C. (1973) Stimulation of Clonogenic Growth of Tumour Cells and Metastases in the Lungs by Local X-irradiation. *Br. J. Cancer*, **27**, 291.

WINN, H. J. (1972) *In Vivo* Methods for the Assessment of Antibody-mediated Tumor Immunity. *Natn. Cancer Inst. Monog.*, **35**, 13.

WITHERS, H. R. & MILAS, L. (1973) Influence of Pre-irradiation of Lung on Development of Artificial Pulmonary Metastases of Fibrosarcoma in Mice. *Cancer Res.*, **33**, 1931.

WOOD, S., HOLYOKE, E. D. & YARDLEY, J. H. (1961) Mechanisms of Metastasis Production by Blood-borne Cancer Cells. *Can. Cancer Conf.*, **4**, 167.

WOODRUFF, M., DUNBAR, N. & GHAFFAR, A. (1973) The Growth of Tumours in T-cell Deprived Mice and Their Response to Treatment with *Corynebacterium parvum*. *Proc. R. Soc. Lond. B*, **184**, 97.